# American Proctological Society

**Published:** 1913-12

**Authors:** 


### American Proctological Society.

The following is an abstract of some of the papers read at June meeting:

### A METHOD OF OPERATING ON FISTULA WITHOUT CUTTING MUSCULAR TISSUE.

BY ROLLIN H. BARNES, M. D., OF ST. LOUIS, MO.

This method is used in those cases of fistula which involve the sphincter muscles. An incision is made external to the sphincter, similar to that made when incising an ischio-rectal abscess.

Through this opening the scar tissue is dissected out up to the internal opening. An incision is then made at the skin margin, so that the middle of this incision passes through an imaginary longitudinal line drawn from the internal opening. A submucous dissection is then channeled out up to the internal opening. Gauze drainage is kept in this until the external wound is healed sufficiently. Then the submucous tract, which remains, is incised under local anesthesia. No muscular tissue having been cut, the function of the sphincters is preserved intact.

### REPORT OF A CASE OF FECAL TUMOR ASSOCIATED WITH HIRSCHSPRUNG'S DISEASE.

BY ALOIS B. GRAHAM, A. M., M. D., OF INDIANAPOLIS, IND.

Dr. Graham reported a case of fecal tumor associated with Hirschsprung's disease, the clinical history of which is unique and exceedingly interesting. The patient, a young French woman, aged 27, stated that she had undergone three abdominal operations for Hirschsprung's disease, or megacolon.

Present illness dates from birth. Not unusual to go a week or ten days without a stool, and then evacuation was produced only by means of enemata.

At the age of 12 her condition was diagnosed as one of pregnancy on account of the vomiting and the appearance of the abdomen.

At the age of 19 she suffered an attack of complete intestinal obstruction, due evidently to fecal tumor. She was operated, and a large fecal tumor was removed from the sigmoid. Six months later she was operated for post-operative adhesions. No resection of the bowel or short-circuiting operation was performed.

At the age of 25 she suffered an attack of complete intestinal obstruction. She was operated, and a large fecal tumor was removed. Patient stated that the bowel was plicated in closing. Wound healed promptly, but she remained in the hospital for three months purely for clinical purposes.

August, 1912, she, for the third time, presented symptoms of complete intestinal obstruction. She had been absolutely constipated for seven days. Abdomen enlarged and everywhere tympanitic except in the lower right quadrant, where there was a dull area corresponding to a large tumor which could be readily palpated. Tumor, a fecal mass, was exceedingly hard and did not pit on pressure. It could be easily moved in every direction throughout the abdomen. Attacks of violent, colicky pains were frequent. Vomiting was persistent, pulse 120, temperature 101 F. Hydrogen peroxide, introduced into the rectum, had no effect on the tumor, but produced excruciating pains over the entire abdomen. Patient consented to operation with the promise exacted that nothing radical be attempted. She requested that fecal tumor be removed, but refused to give her consent to any short-circuiting or resection of the bowel.

Median incision. No adhesions. Fecal tumor in sigmoid. Tumor of "stony" hardness. Its greatest circumference was 19¾ inches, its weight was 64 ounces. The dilatation which was confined to the sigmoid was very marked, the greatest circumference being 20 inches.

Patient made an uneventful operative recovery, and was discharged from the hospital on the tenth day. She gained in weight and appeared to be in the best of health. She experienced no difficulty in procuring daily evacuations with the aid of small doses of cascara.

December 15, 1912, was the date of her last visit to the writer's office. At this time she was doing nicely. Inquiries as to her whereabouts were made and the reports were to the effect that she has returned to France. Information was received the latter part of April that patient had gone to Chicago from Indianapolis. She evidently suffered another attack of intestinal obstruction. She was operated there April 19, 1913, and died three days later.

---

### A FURTHER CONSIDERATION OF SIR CHARLES BALL'S OPERATION FOR INTERNAL HEMORRHOIDS.

BY ALFRED J. ZOBEL, M. D., OF SAN FRANCISCO, CAL.

After a trial of this operation the author of the paper sums up his conclusions as to its value, as follows: That, as a modification of the old ligature operation, it is better than the latter, and at the same time is far superior to the clamp and cautery operation, in that it takes care of and avoids the recurrence of that revolted anal skin ring which generally becomes markedly edematous immediately after these operations, leaving behind skin tags after the swelling subsides.

In every instance in which the essentials of Ball's technique have been followed out carefully the author's results have been exceedingly satisfactory.

The operation is recommended.

---

### DEDUCTIONS BASED ON AN ANALYSIS OF THREE THOUSAND RECTAL CASES.

BY T. CHITTENDEN HILL, M. D., OF BOSTON, MASS.

The principal object of this tabulation of 3000 consecutive rectal cases was to furnish data as to the relative frequency of the various affections of the rectum and colon. There was a total of 1120 operations performed in this series, and some deductions of a practical nature were drawn from this experience. It was found that rectal ailments were more common among males than females, the ratio being three to two.

Hemorrhoids formed a large percentage, 41 per cent of the total. Next in frequency were abscesses and fistulæ, 18 per cent, and the remaining disorders were tabulated as follows: Pruritis ani 18

per cent, anal fissure 10 per cent, colitis 6 per cent, prolapsus ani and procidentia recti 3.7 per cent, cancer of the rectum and sigmoid 2 per cent, benign growths 1.5 per cent, stricture 1.5 per cent, syphilis 2 per cent, constipation 2.8 per cent.

Other miscellaneous conditions were recorded which made up but a fraction of one per cent, such as anal verrucca, congenital stenosis, patulous anus, pilo-nidal sinus, furuncles, foreign body, incontinence, coccygodynia, trauma, sigmoid diverticulitis, etc.

### Z-PLASTIC OPERATION FOR ANAL STRICTURE.

BY WM. M. BEACH, M. D., OF PITTSBURG, PA.

The writer states that extensive cicatrices, resulting from trauma, and involving the partial or entire anal circumference, not infrequently resist the usual methods employed to restore the physiologic function of the anus.

He therefore employed what he terms a Z-plastic method when operating on an anal stricture. The principle underlying the procedure is the transposition of dermic tissue in such manner as to obliterate the crest of the fibrous band.

The first incision is made along the crest of such a band; then incisions are made at right angles from both sides, but running in opposite directions, thus approximating the letter Z. The flaps thus outlined are dissected up, transposed and sutured. Various modifications are permissible, according to the extent of the stricture.

